# White Matter Changes Associated with Resting Sympathetic Tone in Frontotemporal Dementia vs. Alzheimer’s Disease

**DOI:** 10.1371/journal.pone.0142445

**Published:** 2015-11-25

**Authors:** Mario F. Mendez, Simantini J. Karve, Madelaine Daianu, Elvira Jimenez, Paul Thompson

**Affiliations:** 1 Department of Neurology, David Geffen School of Medicine, University of California Los Angeles, 710 Westwood Plaza, Los Angeles, California, 90095, United States of America; 2 Department of Psychiatry & Biobehavioral Sciences, David Geffen School of Medicine, University of California Los Angeles, 710 Westwood Plaza, Los Angeles, California, 90095, United States of America; 3 Imaging Genetics Center, Institute for Neuroimaging & Informatics, University of Southern California, Los Angeles, California, 90033, United States of America; 4 Department of Neurology, University of Southern California, Los Angeles, California, 90033, United States of America; 5 Department of Psychiatry, University of Southern California, Los Angeles, California, 90033, United States of America; 6 Department of Radiology, University of Southern California, Los Angeles, California, 90033, United States of America; 7 Department of Radiology, University of Southern California, Los Angeles, California, 90033, United States of America; Taipei Veterans General Hospital, TAIWAN

## Abstract

**Background:**

Resting sympathetic tone, a measure of physiological arousal, is decreased in patients with apathy and inertia, such as those with behavioral variant frontotemporal dementia (bvFTD) and other frontally-predominant disorders.

**Objective:**

To identify the neuroanatomical correlates of skin conductance levels (SCLs), an index of resting sympathetic tone and apathy, among patients with bvFTD, where SCLs is decreased, compared to those with Alzheimer’s disease (AD), where it is not.

**Methods:**

This study analyzed bvFTD (n = 14) patients and a comparison group with early-onset AD (n = 19). We compared their resting SCLs with gray matter and white matter regions of interest and white matter measures of fiber integrity on magnetic resonance imaging and diffusion tensor imaging.

**Results:**

As expected, bvFTD patients, compared to AD patients, had lower SCLs, which correlated with an apathy measure, and more gray matter loss and abnormalities of fiber integrity (fractional anisotropy and mean diffusivity) in frontal-anterior temporal regions. After controlling for group membership, the SCLs were significantly correlated with white matter volumes in the cingulum and inferior parietal region in the right hemisphere.

**Conclusion:**

Among dementia patients, SCLs, and resting sympathetic tone, may correlate with quantity of white matter, rather than with gray matter or with white matter fiber integrity. Loss of white matter volumes, especially involving a right frontoparietal network, may reflect chronic loss of cortical axons that mediate frontal control of resting sympathetic tone, changes that could contribute to the apathy and inertia of bvFTD and related disorders.

## Introduction

Apathy, inertia, and emotional blunting are core symptoms of behavioral variant frontotemporal dementia (bvFTD), a neurodegenerative disease which also manifests behavioral disinhibition, hyperorality, lack of empathy, compulsive behaviors, and a dysexecutive neuropsychological profile [1]. Apathy, along with social and emotional disengagement, may be the most common manifestations of bvFTD, occurring in from 62 to 89% of these patients [2], and they are also among the most common symptoms of frontal lobe disorders and white matter disease [3]. Apathy, which is a disturbance of motivation involving loss of goal-oriented behavior, interest, and emotional expression [4,5], is associated with decreased physiological arousal and sympathetic tone [6–9]. It is therefore not surprising that patients with bvFTD have decreased resting sympathetic tone [10], a possible contributor to apathy in these patients.

BvFTD is an excellent model for studying the neuroanatomical basis of resting sympathetic tone because of neuropathology in frontal regions involved in sympathetic control and because of the presence of decreased skin conductance levels (SCLs)[10–12]. Skin conductance, which solely depends on sympathetic innervation of the sweat glands, is a readily measurable index of resting sympathetic and physiological arousal [6,13]. Skin conductance measures include SCL, a resting tonic measure reflecting skin conductance lasting up to a minute or more, and skin conductance response (SCR), a reactive stimulus-dependent phasic measure reflecting discrete fluctuation of several seconds. BvFTD patients not only have decreased SCLs, but also tend to demonstrate decreased SCRs to aversive stimuli [10,14]; however, SCL, compared to SCR, is a purer and more sensitive measure of resting sympathetic tone and more reflective of the baseline motivational state of patients with bvFTD [6].

Among patients with bvFTD, we investigated the neuroanatomical correlates associated with decreased resting sympathetic tone as indexed by resting SCL. The bvFTD patients were compared to those with Alzheimer’s disease (AD), which allows for control of dementia variables and for a comparison group without decreased SCLs [10]. We were interested in investigating whether SCL correlated with key gray matter (GM) regions, white matter (WM) volumes, and WM metrics of fiber integrity in tracts affected by frontal regions. The WM metrics were extracted from diffusion tensor imaging (DTI) and describe the WM microstructure and fiber integrity within carefully delineated WM regions of interest (ROIs). We anticipated that frontal GM ROIs and their corresponding WM tracts would correlate with SCL [15] and that a right hemisphere frontoparietal WM network, reported to be dominant for sympathetic function [16–18], would be specifically associated with resting sympathetic tone. The results were supportive of a network involved in sympathetic tone that primarily involves right WM volumes. We discuss potential explanations for these findings.

## Materials and Methods

### Participants and Clinical Evaluation

We recruited participants from the UCLA Neurobehavior Clinic, after ethics board approval from 1.) The Medical Institutional Review Board (IRB), Office for Protection of Research Subjects, University of California at Los Angeles and 2.) Institutional Review Board-C, Associate Chief of Staff for Research, Veterans Healthcare Administration Center, Greater Los Angeles. All participants and their caregivers provided written informed consent to participate in this study. The participants were community-based, moderately impaired dementia patients. They underwent a comprehensive cognitive and neurological evaluation. We excluded patients on beta-blocker medications that could affect peripheral sympathetic activity, as well as patients with any additional medical, neurologic, or psychiatric disorders. The study aimed to control for group differences in medications; however, AD patients were on acetycholinesterase inhibitors or memantine, drugs commonly used in AD.

The bvFTD patients (n = 14) presented with progressive behavioral changes consistent with apathy and inertia, a decline in social interpersonal conduct, emotional blunting, and lack of insight into their disease. Clinical diagnosis of probable bvFTD was based on International Consensus Criteria for bvFTD [1]. We included only bvFTD patients with mild-to-moderate behavioral disturbances that did not require psychoactive medications. In these patients, the diagnosis was further confirmed by the presence of frontal-anterior temporal predominant changes on neuroimaging with fluorodeoxyglucose positron emission tomography (FDG-PET).

Patients with clinically probable AD (n = 19) who had early onset (<65 years) were included as a comparison group for control of dementia variables and non-specific dementia effects on SCLs. The AD patients met the National Institute of Aging-Alzheimer Association criteria for clinically probable AD (definitive AD requires pathological confirmation) [19]. The bvFTD and AD groups were matched based on variables affecting course of dementia such as age of onset, sex, education, disease duration (years since onset) and Mini-Mental State Exam (MMSE) scores [20].

For the assessment of apathy and inertia, the participants’ caregivers were asked to complete the Scale for Emotional Blunting (SEB) on the patient. The SEB is an excellent measure of all three aspects of apathy, including behavioral (auto-activation), interest, and emotional expression [21–23]. The SEB takes approximately 15–30 minutes and scores the items of the three aspects of apathy on a 3-point scale. Items include lacks plans, ambition, desires, or drive as well as lack of interest and emotional reactivity.

### Psychophysiological Assessment

Electrodermal recording devices were attached to the seated participants. SCL was measured by placing disposable electrodes pre-gelled with isotonic jelly (EL 507, Biopac Inc., Goleta, CA) on the palmar surface of the distal phalanges of the index and middle fingers of the right hand of the participants. The participants were instructed to relax, and SCLs were recorded for 5 minutes. The procedure was done at approximately the same time of day (10:30 a.m.) for all participants, who had refrained from caffeine for at least two hours prior to the testing. SCLs were continuously recorded using the Biopac base module (150MP system) and the skin conductance module (GSR 100C) (Biopac Inc, Goleta, CA) and Biopac AcqKnowledge 4.1 software. Acquisition parameters were set to: 5 μS/V, low pass filter 1Hz, and no high pass filter; the sampling rate was 31.25 Hz. SCLs were processed using MATLAB 2006a to obtain values for each second. An average value of SCL for five minutes was further computed for each participant.

### Neuroimaging

In a separate visit, all participants underwent magnetic resonance imaging (MRI) using a standardized protocol on a 1.5T Siemens Avanto MRI scanner at the MRI Research Center at the University of California, Los Angeles. Standard anatomical T1-weighted sequences were collected (256x256 matrix; voxel size = 1x1x1 mm^3^; TI = 900 ms, TR = 2000 ms; TE = 2.89 ms; flip angle = 40), and diffusion-weighted images (DWI) using single-shot multisection spin-echo echo-planar pulse sequence (144x144 matrix; voxel size: 2x2x3 mm^3^; TR = 9800 ms; TE = 97 ms; flip angle = 90 degrees). Thirty-one separate images were acquired for each DTI scan: 1 T2-weighted image with no diffusion sensitization (*b0* image) and 30 diffusion-weighted images (*b* = 1000 s/mm^2^).

#### Preprocessing and coregistration

Non-brain regions were automatically removed from each T1-weighted MRI scan, and from a T2-weighted image from the DWI set using the “Brain Extraction Tool” (BET) provided as a part of the FMRIB Software Library (FSL) developed by the Oxford Centre for Functional Magnetic Resonance Imaging of the Brain (http://fsl.fmrib.ox.ac.uk/fsl/). Anatomical scans subsequently underwent intensity inhomogeneity normalization using the Montreal Neurological Institute’s “nu_correct” tool (www.bic.mni.mcgill.ca/software/). All T1-weighted images were linearly aligned using FSL (with 6 degrees of freedom) to a common space with 1mm isotropic voxels and a 220×220×220 voxel matrix. The DWIs were corrected for eddy current distortions using the FSL tools (http://fsl.fmrib.ox.ac.uk/fsl/). For each subject, the image with no diffusion sensitization was linearly aligned and resampled to a downsampled version of their T1-weighted image (110×110×110, 2×2×2mm). b0 maps were elastically registered to the T1-weighted scan to compensate for susceptibility artifacts (EPI distortions). Images were visually inspected; there were no misalignments or cases where field of view did not cover the full brain (i.e., cropping).

#### Gray matter (GM) analysis

We analyzed GM by looking at the cortical volume/thickness as extracted from FreeSurfer (v. 5.3) [24] (http://surfer.nmr.mgh.harvard.edu/). We extracted 34 suspected and primarily frontal and temporal ROIs per hemisphere, from the Desikan-Killiany atlas [25] from all native space T1-weighted structural MRI scans (Table 1). For each cortical label, or ROI, we extracted the GM volumes as output by FreeSurfer. We manually adjusted the WM/GM boundaries to obtain most reliable GM volume/thickness measures. We also computed the overall brain volume (i.e., brain size) for each subject and used it as a covariate in the statistical analyses.

**Table 1:** Gray Matter Volumes (from the Desikan-Killiany brain atlas [25]) and White Matter Volumes (from the Johns Hopkins University atlas)^A^.

GRAY MATTER ROIs	WHITE MATTER ROIs
Banks of superior temporal sulcus	Angular white matter
Caudal anterior cingulate	Body of corpus callosum
Caudal middle frontal	Cingulate gyrus
Cuneus	Cingulum white matter
Entorhinal*	Cuneus white matter
Fusiform^R^^,^ *	Fornix) / Stria terminalis
Inferior parietal	Genu of corpus callosum
Inferior temporal	Inferior frontal white matter
Isthmus of the cingulate	Inferior fronto-occipital fasciculus
Lateral occipital	Inferior temporal white matter
Lateral orbitofrontal^B^	Lateral fronto-orbital white matter
Lingual	Middle frontal white matter
Medial orbitofrontal^B^	Middle fronto-orbital white matter
Middle temporal	Middle temporal white matter
Parahippocampal	Pontine crossing tract
Paracentral^R^	Post. thalamic radiation and optic radiation
Pars opercularis	Precuneus white matter
Pars orbitalis^L^	Rectus white matter
Pars triangularis^R^	Sagittal stratum
Peri-calcarine	Splenium of corpus callosum
Postcentral	Superior frontal white matter
Posterior cingulate	Superior fronto-occipital fasciculus
Precentral	Superior longitudinal fasciculus
Precuneus	Superior parietal white matter
Rostral anterior cingulate^R^	Superior temporal white matter
Rostral middle frontal	Supramarginal white matter
Superior frontal^B^	Uncinate fasciculus
Superior parietal	
Superior temporal	
Supra-marginal	
Frontal pole	
Temporal pole^R^	
Transverse temporal	
Insula	

ROIs = Volumetric Regions of Interest

^A^ http://cmrm.med.jhmi.edu/cmrm/atlas/human_data/file/AtlasExplanation2.htm

^R,L and B^Indicate Right, Left, or Bilateral significant GM atrophy in bvFTD, vs. AD

*Indicate Right entorhinal and Left fusiform significant GM atrophy in AD, vs. bvFTD

#### White matter (WM) analysis

We extracted 27 WM ROIs per hemisphere for analysis from the Johns Hopkins University (JHU) WM atlas [26] because they involved all frontal and temporal cortical regions (Table 1). We computed the total volume of each WM ROI based on the total number of voxels in each of the ROIs. We computed fractional anisotropy (FA) at each voxel in the brain from the eddy- and EPI- corrected DWI volumes using the dtifit command as part of the FSL toolbox. We registered the FA image from the JHU atlas to each subject using an elastic deformation algorithm [27]. We applied the deformation using nearest neighbor interpolation to the atlas (http://cmrm.med.jhmi.edu/cmrm/atlas/human_data/file/AtlasExplanation2.htm). This helped to avoid intermixing of labels and registered the atlas ROIs in the same space as the DTI volumes. In this way, we computed average FA and mean diffusivity (MD) measures within the delineated boundaries of each ROI for each individual subject.

### Statistical Analysis

Data analysis was conducted using SPSS version 20 (IBM Inc.) for SCL. Where appropriate, we covaried for brain volume, age, and gender and used the False Discovery Rate (FDR) to correct for multiple comparisons. First, we computed an average SCL for the entire time period of five minutes and examined group differences, including demographics, using one-way analysis of variance. We also examined correlations between demographic/clinical variables, SEB scores, and SCL scores. Second, we analyzed the structural differences as depicted by GM, WM, FA, and MD values between the two diagnostic groups using linear regression. Third, we performed partial correlation analyses between SCLs and the GM, WM, FA, and MD results for all subjects, again with FDR corrections for multiple comparisons. Finally, we ran partial correlations for right hemisphere frontoparietal WM tracts reported as implicated in sympathetic control.

## Results

### Clinical and SCL Comparisons

**Fig 1:**
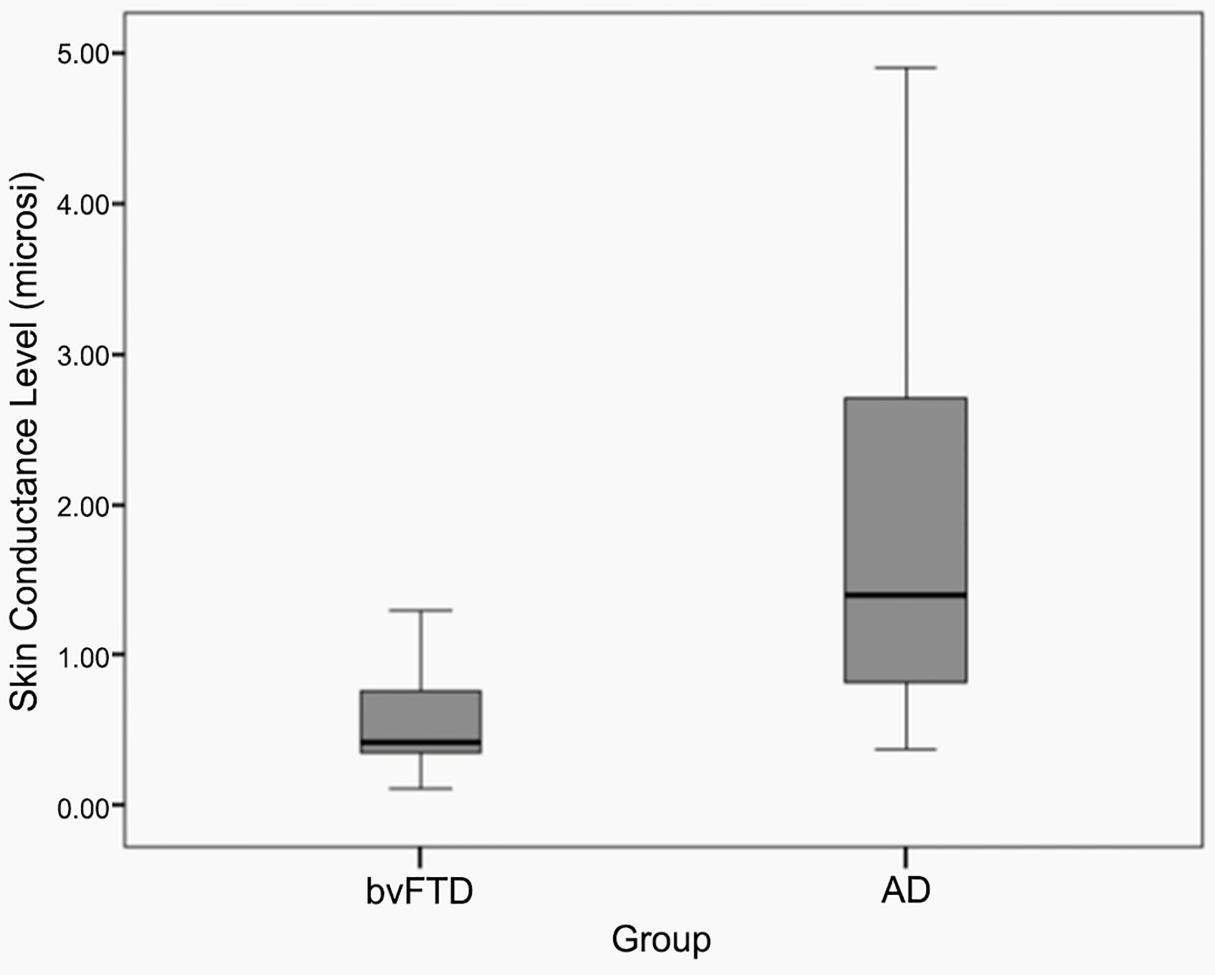
Box plot of Skin Conductance Levels (SCLs) in bvFTD and AD. The dark lines are the means and the boxes and bars indicate interquartile ranges.

There were no significant differences in age, sex, ethnicity, education, disease duration, and MMSE scores between the two groups (Table 2). As expected, the bvFTD patients scored higher on all measures of the SEB, compared to the AD patients (*p*<0.001). Also as expected, the bvFTD patients had significantly lower SCLs as compared to the AD patients (F = 9.09; p < .01). Box plots of SCL differences between the bvFTD and AD groups revealed wide differences in quartile scores (Fig 1). No significant correlations were detected between SCL or SEB scores and age, sex, ethnicity, education, and disease duration within or across both groups. However, there was a significant negative correlation between total SEB and SCL scores (*r* = -0.453; *p*<0.02), i.e., the greater the SEB scores as an indicator of apathetic behaviors, the lower the SCLs.

**Table 2:** Characteristics of the bvFTD and AD patients.

	bvFTD, n = 14	AD, n = 19	Significance
**Age (years)**	62.1 (10.6)	57.9 (4.2)	non-significant
**Sex (Male)**	5; 35.7%	9; 47.3%	non-significant
**Ethnicity (Caucasian)**	12; 85.7%	18; 94.7%	non-significant
**Education**	16 (2.2)	15.9 (1.9)	non-significant
**Disease duration (years)**	3.25 (1.3)	4.0 (2.1)	non-significant
**Mini-Mental State Examination**	24.3 (4.5)	23.5 (4.9)	non-significant
**SEB^A^ Total**	22.2 (8.4)	6.5 (6.1)	F = 32.4; *p <* .001
** ↑apathetic behavior**	9.4 (4.7)	2.9 (3.0)	F = 19.5; *p <* .001
** ↓affect/emotional expression**	6 (2.9)	2.0 (2.3)	F = 15.7; *p <* .001
** ↓“thought” or interest**	6.5 (1.8)	2.0 (1.8)	F = 50.2; *p <* .001
**Resting SCL**^**B**^ **(μS)**	0.58 (0.4)	1.85 (1.3)	F = 9.09; *p <* .01

**A**SEB = Scale for Emotional Blunting

BSCL = Skin Conductance Level

### Neuroanatomical Group Comparisons

#### Gray matter analysis

There were significant differences in GM volume between bvFTD and AD subjects (with bvFTD coded as 1 and AD coded as 0) in a linear regression controlling for age, gender, and brain volume and using FDR to correct for multiple comparison testing. The bvFTD participants had decreased GM volumes, relative to AD, in 10 frontal and 2 adjacent right anterior temporal ROIs (FDR critical *P* = 0.026) (Table 1). In contrast, the AD participants had decreased GM volumes, relative to bvFTD, in left hemisphere fusiform and right hemisphere entorhinal cortex.

#### White matter analysis

No significant bvFTD vs. AD group differences were observed in WM volume ROIs. We ran a linear regression by coding bvFTD as ‘1’ and EOAD as ‘0’ to test for FA and separately MD ROI group differences controlling for age and sex and using FDR to correct for multiple comparison testing. Similar to the GM ROI findings, the bvFTD group, compared to the AD group, had smaller or worse FA values (FDR critical *P* = 0.023) in most frontal and anterior temporal WM and tracts, more in the right hemisphere than the left, and smaller WM and tracts in cingulate and other long fiber tracts and the supramarginal gyrus again primarily on the right (Table 3). Also consistent with the GM and FA results, the bvFTD group, compared to the AD group, had greater or worse MD values (FDR critical *P* = 0.017) in corresponding regions with the prominent exceptions of smaller MD values in the inferior frontal-temporal WM and tracts on the left and the supramarginal gyrus on the left (Table 3). There were no significant associations between the WM measures of volume, FA, and MD when analyzed across the 54 ROIs.

**Table 3:** Significant Fractional Anisotropy (FA) and Mean Diffusivity (MD) White Matter Values for patients with behavioral variant Frontotemporal Dementia (bvFTD) vs. Alzheimer’s Disease (AD) (L = Left Hemisphere; R = Right Hemisphere).

Fractional Anisotropy			Mean Diffusivity (values are E+04)		
Significance	bvFTD	AD	Significance	bvFTD	AD
**Cingulate Gyrus**					
			**L:** (p = 0.0028)	9.7±0.12	9.0±0.73
**R:** (p = 2.2x10^-5^)	0.25±0.03	0.26±0.03	**R:** (p = 0.0014)	11.4±1.12	10.0±1.13
**Cingulum White Matter**					
**R:** (p = 0.0005)	0.17±0.02	0.19±0.02	**R:** (p = 0.006)	11.9±1.32	11.0±1.24
**Corpus Callosum—Body**					
**R:** (p = 4.4x10^-6^)	0.36±0.04	0.38±0.04	**R:** (p = 0.0006)	11.0±1.28	10.0±0.86
**Corpus Callosum—Genu**					
**L:** (p = 0.003)	0.31±0.04	0.33±0.03			
**R:** (p = 1.2x10^-5^)	0.18±0.03	0.23±0.03	**R:** (p = 1.4x10^-5^)	11.2±1.60	9.0±1.02
**Corpus Callosum—Splenium**					
**L:** (p = 4.7x10^-6^)	0.28±0.10	0.42±0.06	**L:** (p = 8.6x10^-5^)	15.1±4.33	11.0±1.47
**R:** (p = 2.2x10^-6^)	0.13±0.03	0.17±0.03			
**Cuneus White Matter**					
**R:** (p = 3.4x10^-5^)	0.17±0.03	0.20±0.02	**R:** (p = 0.0007)	11.9±1.64	10.0±1.36
**Fornix/Stria Terminalis**					
**R:** (p = 3.3x10^-6^)	0.17±0.03	0.22±0.02	**R:** (p = 2.5x10^-6^)	11.3±1.31	9.0±0.84
**Inferior Frontal White Matter**					
**L:** (p = 0.011)	0.32±0.04	0.35±0.03	**R:** (p = 0.0044)	10.9±1.03	12.0±1.41
**Inferior Fronto-occipital Fasciculus**					
**L:** (p = 8.8x10^-5^)	0.21±0.04	0.28±0.05	**L:** (p = 0.0033)	14.0±3.79	10.0±2.89
			**R:** (p = 0.0108)	10.9±1.22	12.0±1.93
**Inferior Temporal White Matter**					
**L:** (p = 0.003)	0.24±0.05	0.29±0.03	**R:** (p = 0.0019)	10.3±1.39	13.0±1.62
**Lateral Fronto-orbital White Matter**					
**L:** (p = 4.8x10^-7^)	0.27±0.04	0.33±0.03	**L:** (p = 1.5x10^-6^)	10.1±1.44	8.0±0.43
			**R:** (p = 0.0084)	11.1±1.10	12.0±1.82
**Middle Frontal White Matter**					
**L:** (p = 0.00067)	0.29±0.04	0.33±0.03	**L:** (p = 0.00037)	9.76±1.32	9.0±0.74
**Posterior Thalamic and Optic Radiations**					
**R:** (p = 0.0001)	0.19±0.02	0.22±0.02			
**Precuneus White Matter**					
**R:** (p = 0.0028)	0.18±0.02	0.20±0.02			
**Rectus White Matter**					
**R:** (p = 0010)	0.24±0.02	0.26±0.02			
**Sagittal Stratum**					
**L:** (p = 7.3x10^-6^)	0.27±0.11	0.42±0.06	**L:** (p = 6.9x10^-65^)	16.0±5.26	11.0±1.34
**R:** (p = 0.0060)	0.23±0.03	0.26±0.03			
**Superior Frontal White Matter**					
**L:** (p = 0.0004)	0.30±0.07	0.37±0.05	**L:** (p = 0.0029)	15.1±3.36	13.0±1.58
**R:** (p = 5.4x10^-7^)	0.13±0.03	0.17±0.02			
**Superior Fronto-occipital Fasciculus**					
**L:** (p = 0.0013)	0.27±0.08	0.35±0.06	**L:** (p = 0.011)	16.2±3.68	14.0±1.96
**R:** (p = 0.00018)	0.14±0.02	0.16±0.02	**R:** (p = 4.9x10^-5^)	11.6±2.43	9.0±0.73
**Superior Longitudinal Fasciculus**					
**R:** (p = 0.00016)	0.11±0.02	0.15±0.02	**R:** (p = 3.2x10^-5^)	11.4±2.07	9.0±0.86
**Superior Temporal White Matter**					
**R:** (p = 0.017)	0.18±0.02	0.19±0.02			
**Supramarginal White Matter**					
			**L:** (p = 0.0059)	9.98±0.83	11.0±1.08
**R:** (p = 0.018)	0.12±0.03	0.14±0.02	**R:** (p = 9.1x10^-5^)	12.6±2.94	10.0±0.94
**Uncinate Fasciculus**					
**R:** (p = 0.00018)	0.15±0.03	0.17±0.02	**R:** (p = 0.0002)	11.3±2.33	9.0±1.023

### Neuroanatomical Correlates of SCLs

There were no significant associations between the GM volume, FA values, or MD values and the SCL scores in either combined groups or each diagnostic group individually. There were no significant within group correlations for the ROI WM volumes’ however, there was a significant SCL correlation with the right hemisphere cingulum WM volume (0.45, *P* = 0,001) (FDR critical *P* = 0.0019).

**Fig 2:**
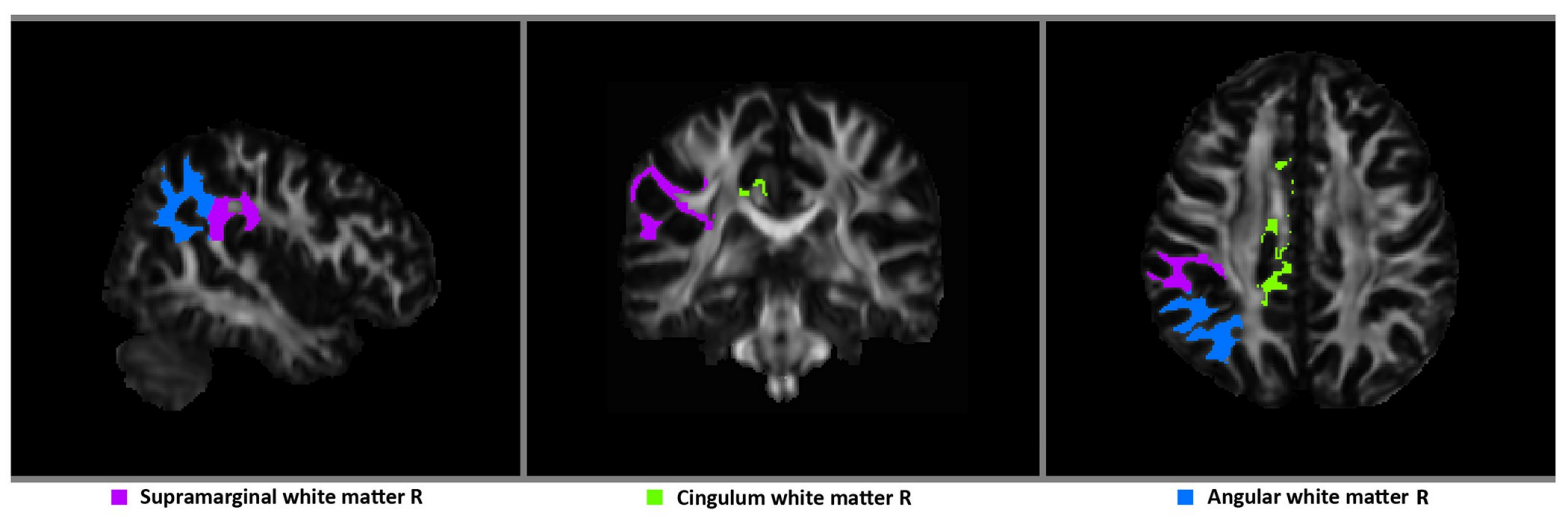
Significant positive correlations of SCLs with white matter (WM) volumes. The 33 bvFTD and AD subjects underwent partial correlations (age, gender, diagnosis) of the SCLs with frontoparietal WM ROIs. R = right; L = left.

To analyze for the presence of a frontoparietal WM network for sympathetic tone, we ran partial correlations across all 33 subjects for right frontoparietal WM (volumes, FA, and MD) implicated in sympathetic control. These areas included superior frontal gyrus, middle frontal gyrus, cingulum, superior and adjacent parietal cortex, including Brodmann’s areas 6,9,10,46 and 7, plus adjacent 39 and 40 [16]. The analysis controlled for age, gender, and group membership. There were no significant findings for FA or MD. There were significant correlations between SCLs and WM volume ROIs in the right cingulum (r = 0.56, *P* = 0,001), right angular gyrus (r = 0.45, *P* = 0,013), and right supramarginal gyrus (r = 0.57, *P* = 0,001) (FDR critical *P* = 0.026) (Fig 2).

## Discussion

Clarifying the basis of decreased resting sympathetic tone suggested correlates for the apathy and inertia that are prominent clinical manifestation of bvFTD and related “frontal” disorders. The bvFTD patients, compared to the AD patients, had lower SCLs, an index of resting sympathetic tone, and greater SEB scores, a measure of apathy, and these measures were inversely correlated. On neuroimaging, the bvFTD patients, compared to the AD patients, had greater GM volume loss and abnormal WM fiber integrity in frontal and right anterior temporal regions. The SCLs, however, did not correlate with these neuroimaging findings, instead they correlated with decreased quantity or volume of WM in right frontoparietal regions. The loss of WM volumes in a right frontoparietal network may mediate decreased frontal cortical control of resting sympathetic tone and contribute to the apathy and inertia of bvFTD and related disorders.

SCL indexes preparation for reactivity or sympathetic tone and is frequently decreased in frontally-predominant brain damaged patients with apathy, a disturbance of motivation with decreased auto-activation and physiological arousal [4,5,23]. These patients usually have damage to frontal cortical regions involved in maintaining a tonic resting level of reactivity. SCL is an excellent psychophysiological measure for evaluating these patients because it is a sensitive and pure measure of tonic baseline sympathetic reactivity and predicts the ease of responsiveness to stimuli [28–32]. In contrast, the much more studied SCR reflects a transient change in SCL caused by a significant or novel stimulus, increases with stress and anxiety, and involves a wider range of brain regions than SCL [33].

Patients with bvFTD, who have high levels of apathy, have volume loss in frontal cortical regions involved in controlling and regulating sympathetic nervous activity, such as the dorsal anterior cingulate cortex (dACC), the ventromedial prefrontal cortex (vmPFC), and the orbitofrontal cortex (OFC) [10,22,34–38]. The neuropathology of bvFTD particularly affects the dACC, which positively correlates with SCLs [33,39], and the vmPFC, which inversely correlates with SCLs [40]. BvFTD also involves other regions that affect SCLs, such as the OFC and the anterior insulae (AI), with its amygdalar connections, particularly on the right [33,41–44]. A more complete understanding of resting sympathetic tone must extend beyond these modular centers to the neural pathways emanating from these frontal regions [39]. Patients with bvFTD have reductions in anterior portions of frontal and temporal white matter, compared to controls [45], and DTI measures of WM fiber integrity are sensitive measures for bvFTD [46–50]. Furthermore, recent studies have emphasized the differential effects of bvFTD on the “salience network” with its ACC-AI hub [15,51]. From these and other studies on the sympathetic nervous system, the structures most implicated in the low SCLs found in bvFTD are the dACC, AI, the vmPFC, along with right hemisphere frontoparietal WM pathways [16,17,52,53].

Despite decreased frontal GM volumes and WM fiber integrity in bvFTD compared to AD, in this study only WM volumes correlated with SCLs. The lack of SCL correlation with GM volumes may be due to a lesser sensitivity of GM to WM measures in bvFTD [50]. The lack of SCL correlation with WM fiber integrity measures may be due to the fact that these measures depend more on astrocytic gliosis, myelin loss, and neuropathological protein deposits than on uncomplicated axonal loss [54]. In chronic Wallerian degeneration, if axons are lost without this associated neuropathology, and the remaining fibers are intact, then there may be more WM volume loss than alterations in DTI measures of fiber integrity. A second factor affecting DTI water diffusion metrics, such as FA and MD, is the degree of intravoxel orientation coherence [54]. FA describes directionally constrained water flow along the axons, and MD describes water diffusion in all directions with WM injury [55]. Hence, these DTI metrics are most helpful with long fibers with anisotropy where water can most easily diffuse in the direction aligned with the internal structure. If there are many crossing fibers resulting in decreased orientation coherence, the voxel diffusion metrics are an average or may represent the primary fiber with the best reconstructed fiber directionality. In other words if there is long-standing Wallerian degeneration, there may be relatively more WM volume loss than abnormalities in FA and MD because the remaining crossing fibers counter some of the altered diffusivity.

This study suggests that decreased WM volumes modulate resting skin conductance in patients with bvFTD. These WM ROIs may group into primarily right hemisphere WM tracts that respond to motivationally-significant stimuli [56]. This is consistent with the finding that the sympathetic nervous system is predominantly controlled by the right side of the brain, while the left side predominantly controls the parasympathetic nervous system [17,18,57]. These descending connections may emanate from the ACC, the AI, and the vmPFC [40,58], the regions that regulate resting state sympathetic tone [15,57,59,60]. The ACC and AI, especially on the right, participate in integrating autonomic bodily states with behavior through sympathetic arousal and reactivity [6,33,57,59,61–63], whereas increased activity in the vmPFC, which occurs with attentional disengagement, decreases SCLs [6]. This study suggests that the combined neuropathology in these three areas, which are particularly affected in bvFTD, results in a net decrease in resting sympathetic tone through decreases in the WM volumes emanating from them.

This study has potential limitations. First, the sample sizes were relatively small. The number of patients/group, however, were large enough to detect significant group differences on the SCLs and neuroimaging measures. Second, this study did not include a normal control group, but it is already known that bvFTD decreases SCL while AD maintains or even elevates it [10]. Here, we studied a comparably demented AD group, to control for confounding variables associated with the presence of dementia, while retaining sufficient variability in SCLs for correlational analysis with DTI findings. Third, we could not control for the fact that the AD patients, and not the bvFTD patients, were on some medications, such as acetylcholinesterase inhibitors and memantine. Finally, our explanation for different sensitivities of WM volume vs. WM DTI metrics of fiber integrity needs further investigation.

In conclusion, we examined the neuroanatomical relationships of SCL in two dementia groups. The SCL correlations suggest loss of right hemisphere WM volumes concerned with mediating resting sympathetic tone presumably from frontal regions regulating the sympathetic nervous system. It is the quantity of WM volume, rather than GM volume or diffusion metrics of WM fiber integrity that reflects disease from frontal lobe sympathetic structures. Much more research is needed to clarify these intriguing, but preliminary, findings and their relationship to clinically important manifestations, such as apathy.

## Supporting Information

S1 File General dataset for deriving information and statistical results reflected in Tables 1, 2 and 3 as well as for Figs 1 and 2. (DAT)

## Abbreviations

AD: Alzheimer’s disease
ACC: anterior cingulate cortex
AI: anterior insula
bvFTD: behavioral variant frontotemporal dementia
DTI: diffusion tensor imaging
FA: fractional anisotropy
MD: mean diffusivity
SCL: skin conductance level
SCR: skin conductance response
vmPFC: ventromedial prefrontal cortex
ROI: region of interest
WM: white matter
